# Impact of hospital mergers on staff job satisfaction: a quantitative study

**DOI:** 10.1186/1478-4491-12-70

**Published:** 2014-12-12

**Authors:** Ka Keat Lim

**Affiliations:** Healthcare Statistics Unit, Clinical Research Centre, National Institute of Health, Ministry of Health Malaysia, 3rd Floor, MMA House, 124 Jalan Pahang, 53000 Kuala Lumpur, Malaysia

**Keywords:** Hospital merger, Hospital restructuring, Job satisfaction, Event study, Difference-in-difference

## Abstract

**Background:**

Hospital mergers began in the UK in the late 1990s to deal with underperformance. Despite their prevalence, there is a lack of research on how such organizational changes affect the staff morale. This study aims to assess the impact of NHS hospital mergers between financial years 2009/10 and 2011/12 on staff job satisfaction and to identify factors contributing to satisfaction.

**Methods:**

Data on staff job satisfaction were obtained from the annual NHS Staff Survey. A list of mergers was compiled using data provided by the Cooperation and Competition Panel and the Department of Health. Other sources of data included the NHS Hospital Estates and Facilities Statistics, the NHS ‘Quarter’ publication, official reports from health service regulators, individual hospitals’ annual accounts, data from the NHS Information Centre and the NHS Recurrent Revenue Allocations Exposition Book. Only full mergers of acute and mental health hospitals were analyzed. Propensity scores were generated using observable factors likely to affect merger decision to select three comparable hospitals for every constituent hospital in a merger to act as a control group. A difference-in-difference was estimated between baseline (3 years before merger approval) and each subsequent year up to 4 years post-merger, controlling for work environment, drivers of job satisfaction, data year, type of hospital and occupation group.

**Results:**

There were nine mergers during the study period. Only job satisfaction scores 1 to 2 years before (0.03 to 0.04 point) and 1 year after merger approval (0.06 point) were higher (*P* < 0.01) than baseline. Robustness testing produced consistent findings. Assuming other conditions were equal, an increase in autonomy, staff support, perceived quality and job clarity ratings would increase job satisfaction scores. Higher job satisfaction scores were also associated with being classified as medical, dental, management or administrative staff and working in a mental health trust.

**Conclusion:**

Hospital mergers have a small, transient positive impact on staff job satisfaction in the year immediately before and after merger approval. Continuous staff support and management of staff expectations throughout a merger may help to increase staff job satisfaction during the challenging period of merger.

**Electronic supplementary material:**

The online version of this article (doi:10.1186/1478-4491-12-70) contains supplementary material, which is available to authorized users.

## Background

Hospital staff job satisfaction has been positively correlated with patients’ experience [1–3] and quality indicators such as hospital-adjusted mortality ratios [4]. Any organizational restructuring would most likely affect job satisfaction levels and potentially compromise the quality of services provided, yet evidence on the impact of hospital mergers on staff satisfaction is surprisingly scarce. Existing literature comprises mainly qualitative case studies of selected mergers [5–8] and official 'how to' documents [9, 10]. Nevertheless, they unambiguously and unanimously highlight the damaging effect of mergers on job satisfaction. Staff might perceive mergers as a breach of the psychological contract (implicit commitments and expectations between employers and employees) when they feel they are not listened to [5], when they have to ‘suffer’ from delays in service development and job uncertainties [7, 11], or when the anticipated benefits of mergers such as training fail to materialize [12]. Cultures of merging organizations might also clash when they have opposing attitudes towards risk [8, 13] or when the culture of one hospital dominates the other [7]. Meanwhile, the only quantitative study [11] lacked a control group so it was not possible to infer a causal relationship between mergers and job satisfaction.

There are limited models explaining factors contributing to job satisfaction. In Bedeian’s and Armenakis’ model [14], job satisfaction is positively correlated to role clarity but negatively correlated with job stress and propensity to leave. This causal model has been validated in acute [14, 15] and mental health hospital [11] settings. Jackson [15] further identified staff participation in the decision-making processes and perceived influence in job role as two other positive contributors to job satisfaction. Job satisfaction is also positively related to work environment including staff trust in the organization and satisfaction with employer obligations [16].

Hospital mergers in England began in 1997 under the 'cooperation and collaboration' policy of the Labour government to merge hospitals trusts (hereafter addressed as 'hospitals') failing to meet their financial or quality targets with one or more better-performing hospitals [17]. The consequence was a large wave of hospital mergers - 112 out of 223 acute hospitals 'disappeared' in the English National Health Service (NHS) between 1997 and 2006 [17]. Recent mergers were also motivated by the rush to meet the April 2014 deadline to achieve autonomous Foundation Trust (FT) status [18]. While the deadline has since been dropped [19], mergers are believed to remain a preferred policy solution for ‘failing’ hospitals over other measures such as appointing a new senior management team or franchising the hospital’s management to the private sector [20].

Therefore, this study was designed to determine the causal impact of hospital mergers on staff job satisfaction and to identify factors contributing to satisfaction during the merger process. The findings of this study could potentially help hospital managers provide better support to their staff through the process of mergers.

## Methodology

### Difference-in-Difference

The randomized controlled trial is the 'gold standard' study design to attribute causal relationships. However, running a randomized controlled trial of hospital mergers is impractical. Existing studies use an econometric method called difference-in-difference (DID) to estimate the effect of a merger on an outcome (for example, hospital quality) [17]. DID derives causal inference from observational data by comparing the intervention group with a synthetic ‘control group’ over multiple time periods.

### Study design

This was a secondary data analysis using multiple data sources. To estimate the DID, a group of never-merged hospitals sharing similar characteristics as the merged hospitals were selected as counterfactuals. Such hospitals were identified using propensity scores (the probability of a hospital undergoing a merger based on a series of observed variables likely to affect mergers), estimated for each financial year mergers occurred, to account for different pools of potential mergers. Each constituent hospital subject to a merger was assigned three never-merged hospitals (selected on the basis of the first to third closest propensity scores) as a control group. The scores were selected so as to be within the common support range (the score range of both merged and never-merged hospitals) [21]. If two or more constituent hospitals shared a common control, the control was only used once. Balancing properties (similarities) were tested to ensure sufficient similarity between merged and never-merged hospitals.

**Figure 1:**
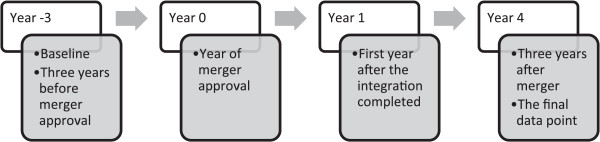
**The study analyzed the impact of hospital mergers on job satisfaction over 8 years.**

As the mergers did not happen in the same year, data were then aligned according to the year the mergers were approved (Figure 1) by the Cooperation and Competition Panel (CCP) [17]. Regression analysis was used to control for confounding factors affecting job satisfaction to estimate the independent effects of a merger. Staff satisfaction 3 years prior to merger approval was used as the baseline because it was most likely to be free from any effect due to anticipation. To assess the robustness of the first matching, the analysis was repeated with two additional sets of controls: fourth to sixth and seventh to ninth hospitals nearest in propensity score.

The study was conducted as part of an MSc at the London School of Economics and Political Science. There was no requirement for ethics approval by the school’s research ethics committee. The usage of NHS Staff Survey data for research was registered with the UK Data Service (usage number 69703) and was subjected to several conditions of use (including maintaining the anonymity of the organizations involved).

### Data sources

Job satisfaction data from the annual NHS Staff Survey were downloaded from the UK Data Service [22–26]. Only datasets from 2008 to 2012 contained identifiable hospital codes. Therefore, the study could only assess mergers between financial years 2009/10 and 2011/12 so that each hospital had at least one pre-merger and one post-merger data point.

The list of mergers was obtained from the CCP’s merger case archive [27] and the Department of Health (DH) in England. Only full mergers involving acute hospitals and mental health hospitals were included in the analysis as they were the main suppliers of secondary care services in England. Part mergers were excluded.

A full list of hospitals and their respective bed numbers was obtained from the NHS Hospital Estates and Facilities Statistics [28]. Annual financial surplus and deficit data for each hospital were compiled from the NHS ‘Quarter’ publication [29], Monitor (the health service regulator for FTs), and individual hospitals’ annual accounts. Annual population mortality data for each Primary Care Trust (PCT, now-defunct administrative bodies responsible for commissioning health services from providers such as hospitals in the English NHS) were obtained from the NHS Information Centre while the volume of services commissioned by PCTs from individual acute hospitals was obtained from the purchaser-provider matrix in the NHS Recurrent Revenue Allocations Exposition Book.

### Selection of control

All merger decisions within the study period were made by the CCP. Hence, three types of variables likely to influence the CCP’s decision were used to generate propensity scores: time varying variables, non-time varying variables and demand.

Time varying variables were bed numbers and annual financial surplus/deficit, each averaged over 2 years prior to merger approval (except a constituent hospital for which only single-year financial data was available) to minimize accidental matching of chronically troubled hospitals with those experiencing transitory problems. The non-time varying variable included the status of the hospital (teaching, mental health and FT). Demand for acute hospitals was proxied by the mortality rate of the population to which actual health services was provided, estimated by weighting the population mortality rate of the PCTs in proportion to the share of services provided by the hospital to each of them. For example, if 30% of total services of Acute Hospital A was commissioned by PCT X and 70% by PCT Y with population mortality rates M and N respectively, the population mortality rate faced by Acute Hospital A would then be 0.3 M + 0.7 N. Similar variables were used by a previous study [17], except that the mortality rate was calculated for a hospital catchment area covering a 30 km radius around the hospital.

### Model specification and variables

The DID regression model (specified below) used the composite job satisfaction score in the NHS Staff Survey as the dependent variable and captured the impact of mergers on job satisfaction score with the DID estimator, δ_3_.

The composite job satisfaction was a simple average of satisfaction scores in eight areas: recognition for good work, support from managers, support from colleagues, freedom to choose their own method of working, the amount of responsibilities given, opportunities to use one’s abilities, the extent to which the employer values one’s work, and the level of pay. Each area was rated based on a 5-point Likert scale, with 1 representing very high dissatisfaction and 5 representing very high satisfaction [30].

Meanwhile, the independent variables (Table 1) were work environment (autonomy, perceived quality of work place, team work, organizational support for staff), drivers of job satisfaction (job clarity, tension) and dummy variables (data year, mental health hospital and occupation group). Each dummy variable takes the value of either 0 or 1 to indicate the presence or absence of a categorical effect that may affect job satisfaction. A detailed definition of each variable is available in Additional file 1.

**Table 1:** **Independent variables for the difference-in-difference (DID) model**

	Categories	Variable name	Descriptions
Work environment	Autonomy	*improve*	Can contribute towards improvements?
		*trusted*	I am trusted to do my job
	Perceived quality of Trust	*satis*	Satisfied with quality of work?
		*toppriority*	Care of patients/service users is my Trust's top priority
		*recomd*	I would recommend my Trust as a place to work
		*incident*	Action taken following errors
	Team work	*discusseff*	Team members often meet to discuss the teams effectiveness
		*teamcomm*	Team members have to communicate closely with each other to achieve the teams objectives
	Support from organization	*equal*	Trust provides equal opportunities to staff?
		*qualapp*	Had good quality appraisal in last 12 months?
		*hands*	Had health and safety training in last 12 months?
		*trainbetter*	My training, learning and development has helped me to do my job better/more effectively
		*upprof*	My training, learning and development has helped me stay up-to-date with professional requirements
		*supsup*	Support from supervisor
		*commun*	Good communication between managers and staff?
Drivers of job satisfaction	Job clarity	*differ*	Role makes a difference?
		*clearobj*	I have, clear, planned goals and objectives for my job
		*knowrep*	I always know what my responsibilities are
	Tension	*wkpres*	Work pressure felt
		*exthrs*	Work extra hours?
		*stress*	Suffered work-related stress in last 12 months?
		*viopat*	Experienced violence from patients/relatives in last 12 months?
		*viocol*	Experienced violence from colleagues in last 12 months?
		*harpat*	Experienced harassment from patients/relatives in last 12 months?
		*harcol*	Experienced harassment from colleagues in last 12 months?
Dummy variables	Data year	*2008*	Dummy variable for year 2008
		*2009*	Dummy variable for year 2009
		*2010*	Dummy variable for year 2010
		*2011*	Dummy variable for year 2011
		*2012*	Dummy variable for year 2012
	Mental health hospital	*mh*	Dummy variable for working in mental health trusts
	Occupation group	*nurses*	Dummy variable for nurses
		*medden*	Dummy variable for medical and dental staff
		*ahp*	Dummy variable for allied health professionals
		*s&t*	Dummy variable for science and technical staff
		*admingm*	Dummy variable for administrative and general management staff

The DID regression did not control for staff demographic characteristics such as age and sex because the data could not be released by data provider due to ethical concerns.


*jobsat* = overall job satisfaction score

*X*_*i*_ = independent variables

*M* = merger dummy: *M* = 1 if the staff worked in a hospital that was selected for merger; *M* = 0 if the staff worked in a hospital that was not selected for merger.

*T* = time period dummy: *T* = 1 for after treatment; *T* = 0 for before treatment

*β*_0_ = y-intercept

*β*_*i*_ = coefficient of independent variables

*δ*_1_ = coefficient of merger dummy, M

*δ*_2_ = coefficient of time period dummy, T

*δ*_3_ = coefficient of interaction term between M and T, also the difference-in-difference (DID) estimator

*e* = error term (assumed independent identically distributed (iid) normal).

### Data analysis

All data analyses were conducted using STATA (StataCorp, College Station, TX, USA) version 11.1 SE*.* Propensity scores were generated using the STATA *pscore* [31] package. Data were managed using Microsoft Access 2010 and Microsoft Excel 2010 (Microsoft Corp., Redmond, WA, USA). As staff from the same hospital might have similar variation of job satisfaction scores, standard errors were clustered by hospitals.

## Results

This section presents the combined analysis of acute and mental health hospitals whose controls were selected using propensity scores generated based on time-varying and non-time varying variables, since mortality rates do not adequately reflect the demand of mental health hospitals. A separate analysis of the demand variable was done on acute hospitals alone, and is presented in Additional file 2 as the results are similar.

### Descriptive findings

**Figure 2:**
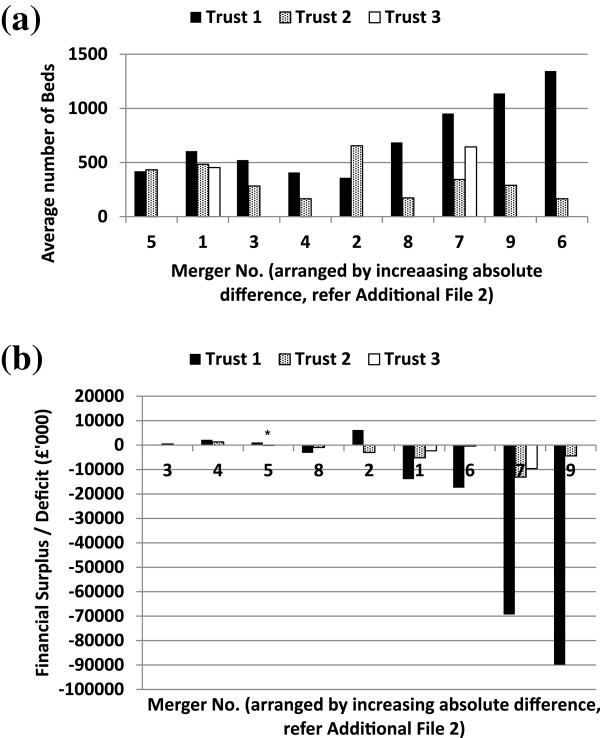
**Bed numbers and financial surplus/deficit of constituent hospitals for each merger.** Only single-year financial data were available for the constituent hospital labeled *. Other data were averaged over 2 years pre-merger. **(a)** Bed numbers **(b)** Financial surplus/deficit.

Nine full mergers were identified during the study period, 7 of which were mergers of acute hospitals (16 constituent hospitals) and 2 of which were mergers of mental health hospitals (4 constituent hospitals) (see Additional file 3). The constituent hospitals were either small or had large differences in size (Figure 2a); the majority (14) were in deficit prior to merger (Figure 2b).

Five acute hospital mergers had one post-merger data point; two acute hospital mergers had four; one mental health hospital merger had one; another mental health hospital merger had three. Aligning them by the year of merger approval (Table 2), merged and control hospitals had 30,995 and 152,409 individual staff survey responses respectively. After merger (Year 1 onwards), the number of staff responses reduced as sampling was done from one entity (the merged hospital) as opposed to two or three constituent hospitals.

**Table 2:** **Breakdown of the number of hospital trusts and individual responses**

Event Year, t	(1) Merged Hospitals				(2) Control Hospitals					
	Mental Health		Acute		Mental Health		Acute		Acute ^a^	
	Hos ^b^	Id ^c^	Hos ^b^	Id ^c^	Hos ^b^	Id ^c^	Hos ^b^	Id ^c^	Hos ^b^	Id ^c^
−3	2	918	11	3,958	6	2,170	23	9,653	21	8,973
−2	2	913	11	4,243	6	2,140	23	9,261	21	8,833
−1	4	1,764	11	4,532	10	4,577	23	9,043	21	8,758
0	4	1,693	16	6,315	10	4,674	28	13,330	28	13,199
1	2	931	7	2,707	10	4,731	28	12,805	28	12,698
2	1	446	2	742	6	2,361	9	3,495	10	3,994
3	1	400	2	733	6	2,210	9	3,716	10	4,096
4	0	0	2	700	0	0	9	3,634	10	4,058

^a^Selected using propensity score estimated by taking into account population mortality rate faced by merged hospitals.

^b^Number of hospitals.

^c^Number of individual staff responses.

Id, individual response; Hos, hospital trust.

### Impact of merger on job satisfaction

Balancing properties (similarities between merged and never-merged hospitals) were fulfilled in all merger years. Controlling for work environment, drivers of job satisfaction and dummy variables, job satisfaction scores were higher in all years compared to baseline until Year 3. However, only the differences at Year −2 (0.03 point), Year −1 (0.04 point) and Year 1 (0.057 point) achieved statistical significance. Robustness tests (Table 3) produced consistent results with one small difference - the DID estimator at Year −2 was no longer statistically significant, possibly because the control hospitals were less similar to the merged hospitals (≥0.1 difference in propensity score) relative to those selected for the main analysis (≤0.015 difference) (Table 3). A separate analysis with acute hospitals alone yielded similar findings (see Additional file 2).

**Table 3:** **Difference-in-difference (DID) estimator,** δ**3 in the main analysis and robustness test using Year −3 as baseline**

Year, t	(1) Main analysis	(2) Robustness test	
	**First to third closest PS**	**Fourth to sixth closest PS**	**Seventh to ninth closest PS**
−2	0.027^a^	0.019	0.025
	(0.013)	(0.013)	(0.013)
−1	0.035^b^	0.033^b^	0.042^b^
	(0.012)	(0.012)	(0.012)
0	0.021	0.006	0.018
	(0.012)	(0.013)	(0.013)
1	0.057^b^	0.043^b^	0.051^b^
	(0.015)	(0.015	(0.016)
2	0.003	0.014	0.002
	(0.025)	(0.022)	(0.028)
3	0.031	0.037	0.049
	(0.027)	(0.029)	(0.026)
4	−0.019	−0.030	−0.017
	(0.036)	(0.038)	(0.037)

Note: cluster robust standard errors in parentheses. Baseline: 3 years before merger approval by the regulator.

^a^ *P* < 0.05; ^b^ *P* < 0.01.

PS, propensity score.

### Independent variables affecting job satisfaction

Table 4 presents the coefficients for independent variables in the 3 years during which mergers had statistically significant impact. Variables that have the largest coefficients are related to autonomy (ability to contribute towards improvements and being trusted to do one’s job) and organizational staff support (support from supervisor and equal opportunities). Holding all other variables constant, an increase in 1 unit for either one of these variables is associated with a 0.2 point rise in job satisfaction score. Other significant positive predictors of job satisfaction were perceived quality (satisfaction with quality of work), organizational staff support (good communication with managers and good quality appraisals), job clarity (having clear goals and objectives for one’s job, and an awareness of one’s own responsibilities) and working in a mental health hospital. Meanwhile, all variables related to work tension except working extra hours were negative predictors of job satisfaction, with the strongest being harassment from colleagues.

**Table 4:** **Coefficients of independent variables in regressions that showed statistically significant difference in** δ_**3**_ **term**

	Category	Independent variables ^a^	Year −2	Year −1	Year 1
Work environment	Autonomy	*improve*	0.187^c^	0.199^c^	0.218^c^
			(0.007)	(0.009)	(0.008)
		*trusted*	0.172^c^	0.151^c^	0.137^c^
			(0.007)	(0.007)	(0.007)
	Perceived quality	*satis*	0.035^c^	0.049^c^	0.042^c^
			(0.011)	(0.010)	(0.011)
		*recomd*	0.115	0.113	0.111^c^
			(0.006)	(0.007)	(0.006)
		*incident*	0.035	0.040	0.040^c^
			(0.009)	(0.009)	(0.008)
	Team work	*discusseff*	−0.078^c^	0.006	0.008
			(0.009)	(0.005)	(0.006)
		*teamcomm*	−0.039^b^	0.036^c^	0.013
			(0.015)	(0.007)	(0.008)
	Support from organization	*equal*	0.169^c^	0.172^c^	0.176^c^
			(0.013)	(0.015)	(0.013)
		*qualapp*	0.082^c^	0.089^c^	0.095^c^
			(0.007)	(0.008)	(0.007)
		*upprof*	0.007	0.012^b^	0.006
			(0.006)	(0.006)	(0.006)
		*supsup*	0.217^c^	0.225	0.242^c^
			(0.006)	(0.006)	(0.006)
		*commun*	0.113^c^	0.112^c^	0.109^c^
			(0.009)	(0.009)	(0.008)
Drivers of job satisfaction	Job clarity	*clearobj*	0.062^c^	0.056^c^	0.057^c^
			(0.006)	(0.006)	(0.005)
		*knowrep*	0.057^c^	0.076^c^	0.048^c^
			(0.006)	(0.007)	(0.007)
	tension	*wkpres*	−0.080^c^	−0.078^c^	−0.076^c^
			(0.006)	(0.006)	(0.006)
		*exthrs*	0.013	0.034^c^	0.030^c^
			(0.008)	(0.008)	(0.008)
		*stress*	−0.085^c^	−0.067^b^	−0.078^c^
			(0.008)	(0.008)	(0.008)
		*viopat*	−0.063^c^	−0.054^c^	−0.062^c^
			(0.012)	(0.011)	(0.011)
		*harpat*	−0.034^c^	−0.049^c^	−0.027^c^
			(0.008)	(0.008)	(0.008)
		*harcol*	−0.111^c^	−0.114^c^	−0.114^c^
			(0.010)	(0.010)	(0.008)
Dummy variables	Mental health hospital	*mh*	0.068^c^	0.079^c^	0.079^c^
			(0.014)	(0.013)	(0.012)
	Occupation	*medden*	0.022	0.032^b^	0.049^c^
			(0.016)	(0.015)	(0.011)
		*s&t*	-	−0.043^b^	−0.054^b^
			-	(0.020)	(0.021)
		*admingm*	0.022	0.044^c^	0.034^b^
			(0.014)	(0.016)	(0.013)
		R-squared	0.664	0.672	0.679
		Observations	14,639	15,399	16,492

Note: cluster robust standard errors in parentheses. Baseline: 3 years before merger was approved by the regulator. Only statistically significant variables are displayed in the table.

^a^Definitions for each independent variable are shown in Table 1 (also see Additional file 1).

^b^ *P* < 0.05.

^c^ *P* < 0.01.

## Discussion

This study identified 9 mergers of acute and mental health hospitals between financial years 2009/10 and 2011/12. Besides the transient increase in staff job satisfaction score immediately before and after merger approval, the scores in other time periods were not significantly different from the baseline. Selection of control hospitals was shown to be robust. The analysis also identified independent variables contributing to staff job satisfaction during mergers.

### Recent versus previous wave of mergers

Recent mergers were homogenous as all of them were motivated by a broader policy requiring NHS hospitals to achieve FT status by 2014. Nevertheless, the frequency of recent mergers is much lower than the previous wave (1997 to 2006), when there were 20 acute hospitals mergers annually, even involving mergers of large hospitals [17]. This is not surprising because the current government is not actively pursuing merger policy unlike the previous Labour government. In addition, merger decisions have now been delegated to the independent CCP, which focuses in particular on assessing the impact of a proposed merger on competition. For example, merging two large hospitals serving the same population would significantly reduce competition and is against the Principles and Rules for Cooperation and Competition (PRCC) [32].

### Impact of merger on staff job satisfaction

The neutral overall effect of mergers is unexpected as a literature search retrieved solely negative findings about the impact of mergers on staff morale. One possible interpretation is that staff might have ‘adapted’ to regular work place restructuring, and expect recurrent major organizational changes due to frequent health system reforms in the UK [5]. Another explanation is the difference in reference point - the present study compared changes in staff job satisfaction against the baseline at Year −3 as opposed to qualitative studies [6, 7] that interviewed staff during or immediately after a merger. The latter would naturally elicit more negative responses because staff would likely compare their current experiences to the transient peak of job satisfaction prior to merger.

The differential anticipation effect of mergers on different occupation groups has been discussed in Corrigan *et al*. [9]. As managers and administrative staff are likely behind the planning and execution of a merger, they might perceive a higher chance of success and ensuing resource savings. Meanwhile, medical and dental staff might view a merger as an opportunity to improve their professional standing and to share good practice [7]. Such optimism may be absent among nurses and other health professionals.

### Stages of staff merger experience

**Figure 3:**
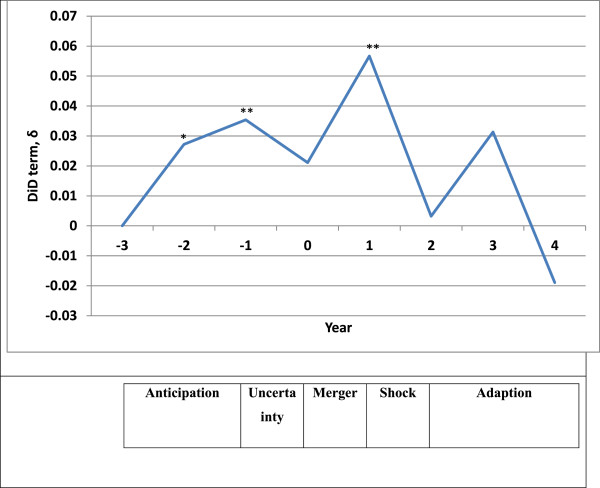
**Five stages of staff merger experience based on fluctuations of δ**_**3**_ **term.** Note: Baseline: 3 years before merger was approved by the regulator. *p<0.05 **p<0.01.

The fluctuations of the DID estimators can be used to divide staff merger experience into five stages: anticipation, uncertainty, merger, shock and adaptation (Figure 3).

The anticipation stage begins as early as 2 years before merger approval when staff first learn of the possibility of a merger. Most of them acknowledge the necessity of organizational reconfiguration to improve patient services and the hospital’s performance [8]. The anticipation driven by staff engagement through pre-merger consultations keeps the job satisfaction higher than baseline for a period of 2 years.

A period of uncertainty follows as it becomes unclear whether the merger will actually proceed, pending a decision by the CCP. Staff begin to worry about loss of managerial control and job security [7], leading to a decrease in job satisfaction. These uncertainties are removed once the regulator gives the 'green light'. Anticipation of benefits and staff engagement again push the job satisfaction above baseline.

Soon after the merger is completed, staff enter a stage of shock as the benefits commonly communicated during pre-merger consultations (for example, improved services to patients and career prospects [6]) fail to materialize. Merged hospitals experience delays in the appointment of middle managers, service development and implementation of the proposed changes [7] and fail to achieve economies of scale as constituent hospitals continue to work in silos [12]. Unmet expectation causes job satisfaction to plunge within a year after integration, similar to a previous observation [11].

As staff adapt and move on, job satisfaction rebounds and remains around baseline. A previous study also find that by the third year post-merger, staff experience less anxiety and stress [7].

### Policy implications

These findings have several policy implications. The statistically significant, yet small, increase in job satisfaction immediately before and after merger approval indicates the success of pre-merger staff engagement. Therefore, such efforts should be continued and possibly intensified throughout the merger process, for example at Year 2, to prevent the sharp decline in job satisfaction that could compromise service quality. This also highlights the importance of managing staff expectations to minimize any post-merger ‘shock’. It is possible that hospital managers underestimate the difficulties in implementing the change process, leading to overstatement of benefits and unrealistic expectations that are later unmet.

The increase in job satisfaction score is small and transient, which might explain the stagnation of health care service quality after a merger [17]. As staff job satisfaction is positively correlated with health care quality [4], a higher job satisfaction and a rise in quality may ensue if staff expectations of the benefits of a merger are met.

### Limitations

Several limitations merit discussion. First and foremost, the generalizability of the findings is hampered by the small number of mergers within the study period. As each merger only has 5 data points, aligning them by stages of merger means that data for certain years contributed few mergers (for example, only 2 mergers at Year 4). In addition, the number of staff survey responses also decreased after a merger due to fewer hospitals from which staff were sampled. This might lead to biased findings.

The analyses were also unable to exclude and to quantify response and non-response bias, as the response rates of the NHS staff survey at hospital level are unknown. Similarly, the datasets have an overall 3.3% of missing data (ranging from 0% to 30% per variable). Conditional imputation was considered but not done due to lack of staff demographic characteristics and lack of an established model to explain how different perceptions affect each other. The analysis adopted the complete case approach in view of the large number of individual responses.

The validity of the results relies heavily on the appropriateness of the control hospitals selected, based on propensity scores. In particular, there was no demand variable to guide the selection of control for mental health hospitals. Nevertheless, robustness checks and a separate analysis using a demand variable for acute hospitals confirmed the main findings that mergers exerted transient positive effects on job satisfaction, but the observations at Year −2 become less conclusive.

Besides that, the study assumed that environmental factors and drivers of job satisfaction were properly controlled for, which is unlikely given the limited number of variables available. The regression model also assumed no reverse causality.

While statistically significant differences were observed, it is difficult to translate the small magnitude of score improvement to actual staff motivation. Finally, it should be noted that DID measured the average treatment effect on the treated rather than the average treatment effect.

## Conclusion

In a nutshell, mergers have a small, transient positive impact on staff job satisfaction immediately before and after a merger. This is associated with autonomy, staff support, perceived quality, job clarity, being in a medical, dental, management and/or administrative role and working in a mental health trust. However, the increase in job satisfaction scores is not sustained and returns to the baseline level within 1 year. While the small magnitude of improvement may not substantiate any drastic policy change, the analysis indicates that continuous staff engagement after a merger and effective management of staff expectations may help to increase and sustain job satisfaction during the merger process.

## Authors’ information

The author conducted the study as part of his MSc International Health Policy (Health Economics) degree programme at the London School of Economics and Political Science (LSE).

## Electronic supplementary material

Additional file 1: **Variable definitions.** definition of all independent variables used in the difference-in-difference model. Some variables were aggregate scores of several questions in the NHS Staff Survey whereas some were scores for individual survey questions. (PDF 143 KB)

Additional file 2: **Separate analysis of acute hospital data.** results of the separate analysis of acute hospitals. The controls were selected using propensity score generated with time-varying, non-time varying and demand variables. (PDF 71 KB)

Additional file 3: **List of mergers.** list of mergers between 2009 to 2012 with their respective approval and merger years. (PDF 68 KB)

## Abbreviations

CCP: Cooperation and Competition Panel
DH: Department of Health
DID: Difference-in-difference
FT: Foundation Trust
NHS: National Health Service
PCT: Primary Care Trust
PRCC: Principles and Rules for Cooperation and Competition.

## References

[1] Borrill C, West M, Carter M, Dawson J (2003). The Relationship Between Staff Satisfaction and Patient Satisfaction: Results from Wolverhampton Hospitals NHS Trust.

[2] Dawson J (2009). Health and Wellbeing of NHS Staff - A Benefit Evaluation Model.

[3] Peltier J, Dahl A, Mulhern F (2009). The Relationship between Employee Satisfaction and Hospital Patient Experiences: Forum for People Performance Management & Measurement.

[4] Pinder RJ, Greaves FE, Aylin PP, Jarman B, Bottle A (2013). Staff perceptions of quality of care: an observational study of the NHS Staff Survey in hospitals in England. BMJ Qual Saf.

[5] Cortvriend P (2004). Change management of mergers: the impact on NHS staff and their psychological contracts. Health Serv Manage Res.

[6] Fulop N, Protopsaltis G, Hutchings A, King A, Allen P, Normand C, Walters R (2002). Process and impact of mergers of NHS trusts: multicentre case study and management cost analysis. BMJ.

[7] Fulop N, Protopsaltis G, King A, Allen P, Hutchings A, Normand C (2005). Changing organisations: a study of the context and processes of mergers of health care providers in England. Soc Sci Med.

[8] Shaw J (2002). Tracking the merger: the human experience. Health Serv Manage Res.

[9] Corrigan P, Higton J, Morioka S (2012). Takeover: Tackling Failing NHS Hospitals.

[10] Health Education Authority (1999). Healthy Ever after? Supporting Staff Through Merger and Beyond.

[11] Gulliver P, Towell D, Peck E (2003). Staff morale in the merger of mental health and social care organizations in England. J Psychiatr Ment Health Nurs.

[12] Goddard S, Palmer A (2010). An evaluation of the effects of a National Health Service Trust merger on the learning and development of staff. Hum Resour Dev Int.

[13] Hofstede G: **Dimensionalizing cultures: The Hofstede Model in context.** *Online Readings Psychol Cult* 2011.,**2**(1)**:** http://scholarworks.gvsu.edu/cgi/viewcontent.cgi?article=1014&context=orpc

[14] Bedeian AG, Armenakis AA (1981). A path-analytic study of the consequences of role conflict and ambiguity. Acad Manage J.

[15] Jackson SE (1983). Participation in decision making as a strategy for reducing job-related strain. J Appl Psychol.

[16] Fielden S, Whiting F (2007). The psychological contract: is the UK National Health Service a model employer?. Health Serv Manage Res.

[17] Gaynor M, Laudicella M, Propper C (2012). Can governments do it better? Merger mania and hospital outcomes in the English NHS. J Health Econ.

[18] House of Commons Committee of Public Accounts (2011). Achievement of Foundation Trust Status by NHS Hospital Trusts: Sixth Report of Session 2010–12.

[19] Department of Health (2013). 2012–13 Update on Indicators of Financial Sustainability in the NHS.

[20] Ham C, Dixon A (2012). Tackling the problems of seriously challenged NHS providers. BMJ.

[21] Stuart EA (2010). Matching methods for causal inference: a review and a look forward. Stat Sci.

[22] Healthcare Commission and Aston University. Aston Business School (2008). National Health Service National Staff Survey, 2008.

[23] Care Quality Commission and Aston University. Aston Business School (2009). National Health Service National Staff Survey, 2009.

[24] Care Quality Commission and Aston University. Aston Business School (2010). National Health Service National Staff Survey, 2010.

[25] Care Quality Commission and Picker Institute Europe (2011). National Health Service National Staff Survey, 2011.

[26] Care Quality Commission and Picker Institute Europe (2012). National Health Service National Staff Survey, 2012.

[27] **Co-operation and Competition Panel case archive** [http://live.monitor.precedenthost.co.uk/regulating-health-care-providerscommissioners/cooperation-and-competition/archive-co-operation-and--5]

[28] **Hospital Estates and Facilities Statistics** [http://hefs.hscic.gov.uk/]

[29] **The Quarter** [http://webarchive.nationalarchives.gov.uk/20130107105354/http://www.dh.gov.uk/en/Publicationsandstatistics/Publications/PublicationsStatistics/DH_087335]

[30] NHS Staff Survey Coordination Centre (2012). Guidance for the NHS Staff Survey 2012.

[31] Becker SO, Ichino A (2002). Estimation of average treatment effects based on propensity scores. STATA J.

[32] Department of Health (2010). Principles and Rules for Cooperation and Competition.

