# Supplementary material for: Impact of hospital mergers on staff job satisfaction: a quantitative study
Source: Hum Resour Health. 2014 Dec 12;12:70. doi: 10.1186/1478-4491-12-70 (PMC4277837; doi:10.1186/1478-4491-12-70)
Supplement: Supplementary file 1 — Additional file 1: Variable definitions. definition of all independent variables used in the difference-in-difference model. Some variables were aggregate scores of several questions in the NHS Staff Survey whereas some were scores for individual survey questions. (PDF 143 KB) [file 12960_2014_462_MOESM1_ESM.pdf]

Additional File 1

Title : Variable definitions

Some of the independent variables used in the DiD regressions were aggregate scores generated in the NHS Staff Survey (named 'Key Findings') whereas some were individual survey questions. This section indicates the types and provides detailed definitions for each of the independent variables

1. *Independent variables which are "Key Findings" in NHS Staff Survey*

Variable Name	Definitions
satis	**Satisfied with quality of work?**<br>Whether staff agreed or strongly agreed with at least two of the following three statements:<br>• I am able to do my job to a standard I am personally pleased with<br>• I am satisfied with the quality of care I give to patients / service users<br>• I am able to deliver the patient care I aspire to
differ	**Role makes a difference?**<br>Whether staff agreed or strongly agreed with the statement:<br>• I feel that my role makes a difference to patients / service users
wkpres	**Work pressure felt**<br>Average rating (each rated on a 1 – 5 scale) for the following statements:<br>• I have adequate materials, supplies and equipment to do my work.<br>• There are enough staffs at this Trust for me to do my job properly.<br>• I cannot meet all the conflicting demands on my time at work.
exthrs	**Work extra hours?**<br>In an average week, whether they work longer than the hours for which they are contracted.
qualapp	**Had good quality appraisal in last 12 months?**<br>Whether staff had an appraisal in the previous 12 months and answered "yes" to each of the following three questions:

	• Did it help you to improve how you do your job?<br>• Did it help you agree clear objectives for your work?<br>• Did it leave you feeling that your work is valued by your organisation?
supsup	**Support from supervisor**<br>Average rating (each rated on a 1 – 5 scale) for the following statements:<br>• My supervisor encourages those who work for her or him to work as a team.<br>• My supervisor can be counted on to help me with a difficult task at work.<br>• My supervisor gives me clear feedback on my work.<br>• My supervisor asks for my opinion before making decisions that affect my work.<br>• My supervisor is supportive in a personal crisis.
hands	**Had health & safety training in last 12 months?**<br>Whether staff who had received health and safety training paid for or provided by their trust, in the last 12 months
stress	**Suffered work-related stress in last 12 months?**<br>Whether staff had felt unwell as a result of work related stress in the last 12 months
incident	**Action taken following errors**<br>Average rating (each rated on a 1 – 5 scale) for the following statements<br>• My Trust treats staffs who are involved in an error, near miss or incident fairly.<br>• My Trust encourages us to report errors, near misses or incidents.<br>• My Trust treats reports of errors, near misses or incidents confidentially.<br>• My Trust blames or punishes people who make errors, near misses or incidents.<br>• We are informed about errors, near misses and incidents that happen in the Trust.<br>• We are given feedback about changes made in response to reported errors, near misses and incidents.<br>• When errors, near misses or incidents are reported, my Trust takes action to ensure that they do not happen again.
violpat	**Experienced violence from patients/relatives in last 12 months?**<br>Whether staff experienced physical violence from patients / service users, their relatives or other members of the public in the previous 12 months
violcol	**Experienced violence from colleagues in last 12 months?**<br>Whether staff experienced physical violence from colleagues or managers in the previous 12 months.

harpat	**Experienced harassment from patients/relatives in last 12 months?**<br>Whether staff had experienced harassment, bullying or abuse at work from patients / service users, patients / service users, their relatives or other members of the public in the previous 12 months.
harcol	**Experienced harassment from colleagues in last 12 months?**<br>Whether staff experienced harassment, bullying or abuse from colleagues or managers in the previous 12 months.
commun	**Good communication between managers and staff?**<br>Whether staff agreed or strongly agreed with at least three of the following four statements:<br>• Senior managers here try to involve staff in important decisions<br>• Communication between senior management and staff is effective<br>• I know who the senior managers are here<br>• Senior managers act on staff feedback
improve	**Can contribute towards improvements?**<br>Whether staff agreed or strongly agreed with at least two of the following three statements:<br>• There are frequent opportunities for me to show initiative in my role<br>• I am able to make suggestions to improve the work of my team / department<br>• I am able to make improvements happen in my area of work
equal	**Trust provides equal opportunities to staff?**<br>Whether staff said that their trust acts fairly with regards to career progression / promotion, regardless of ethnic background, gender, religion, sexual orientation, disability or age.

2. Independent variables which are individual questions in NHS Staff Survey

These variables are in the form of rating on scale of 1 – 5 each.

Variable Name	Definitions
discusseff	Team members often meet to discuss the teams effectiveness
teamcomm	Team members have to communicate closely with each other to achieve the teams objectives
trainbetter	My training, learning and development has helped me to do my job better / more effectively
upprof	My training, learning and development has helped me stay up-to-date with professional requirements
toppriority	Care of patients / service users is my Trusts top priority.
recomd	I would recommend my Trust as a place to work.
clearobj	I have, clear, planned goals and objectives for my job.
knowrep	I always know what my responsibilities are.
trusted	I am trusted to do my job.
