# Supplementary material for: Impact of hospital mergers on staff job satisfaction: a quantitative study
Source: Hum Resour Health. 2014 Dec 12;12:70. doi: 10.1186/1478-4491-12-70 (PMC4277837; doi:10.1186/1478-4491-12-70)
Supplement: Supplementary file 2 — Additional file 2: Separate analysis of acute hospital data. results of the separate analysis of acute hospitals. The controls were selected using propensity score generated with time-varying, non-time varying and demand variables. (PDF 71 KB) [file 12960_2014_462_MOESM2_ESM.pdf]

Additional File 2

Title : Separate Analysis of Acute Hospital Data

Results of the separate analysis of acute hospitals. The controls were selected using propensity score generated with time-varying, non-time varying and demand variables.

Difference-in-difference estimator δ3 using data from acute trust mergers alone.

Year, t	(1) Main Analysis	(2) Robustness Test	
	1st – 3rd Closest PS	4th – 6th Closest PS	7 – 9th Closest PS
**-2**	0.022	0.028	0.038
	(0.016)	(0.017)	(0.015)
**-1**	0.040**	0.043**	0.042**
	(0.012)	(0.011)	(0.012)
**0**	0.014	0.013	0.018
	(0.013)	(0.012)	(0.013)
**1**	0.048**	0.053**	0.065**
	(0.018)	(0.018)	(0.019)
**2**	0.023	0.013	0.021
	(0.039)	(0.037)	(0.040)
**3**	0.025	0.030	0.028
	(0.037)	(0.038)	(0.038)
**4**	-0.011	-0.017	-0.011
	(0.035)	(0.038)	(0.036)

Note: Cluster robust standard errors in parentheses. Baseline: 3 years before merger is approved by the regulator.

** p<0.05*

***p<0.01*
