# Supplementary material for: Impact of hospital mergers on staff job satisfaction: a quantitative study
Source: Hum Resour Health. 2014 Dec 12;12:70. doi: 10.1186/1478-4491-12-70 (PMC4277837; doi:10.1186/1478-4491-12-70)
Supplement: Supplementary file 3 — Additional file 3: List of mergers. list of mergers between 2009 to 2012 with their respective approval and merger years. (PDF 68 KB) [file 12960_2014_462_MOESM3_ESM.pdf]

Additional File 3

Title : List of mergers

List of mergers between 2009 – 2012 with their respective approval and merger years

No.	Type of Constituent Trusts	Number of Constituent Trusts	Approval Year	Merger Year
**1**	Acute	3	2009	2009
**2**	Acute	2	2009	2009
**3**	Mental health	2	2009	2010
**4**	Mental health	2	2011	2012
**5**	Acute	2	2011	2012
**6**	Acute specialist & Acute teaching	2	2011	2011
**7**	Acute & Acute teaching	3	2011	2012
**8**	Acute	2	2012	2012
**9**	Acute & Acute teaching	2	2012	2012
